# Chicago Medical Society

**Published:** 1887-08

**Authors:** 


### CHICAGO MEDICAL SOCIETY.

[Official Report.]

STATED MEETING, June 20, 1887.

The President, W. T. Belfield, M. D., in the chair.

Dr. Harold N. Moyer and Dr. Alfred Hinde presented a paper on

"PERIODICALLY RECURRING OCULO-MOTOR PARALYSIS,"

with the report of a case.

The patient had suffered from her seventh year with complete paralysis of the third nerve in all its branches. The attacks recurred at varying intervals of from four to six months and were always accompanied by headache and vomiting. The attacks have grown more frequent of late and since the earlier ones recovery from the paralysis has not been complete.

The literature of the affection was given, and mention made of some sixteen cases so far reported. The etiology, pathology and prognosis also received consideration.

DISCUSSION.

Dr. W. F. Coleman: This paper is so excellent and unique, I certainly have nothing like it in my own experience to relate. I have perhaps fifteen or twenty text-books in English, German and American, but none of them refer to periodical paralysis of the motor oculi nerve, and I have seen no reference to it in any work on the eye. Isolated paralysis of the whole third nerve, even of organic origin, is a rare affection without involving other cerebral nerves. As the interesting point in the paper is the source and pathology of the disease, I will not undertake to discuss any other point. These cases are more interesting, if possible, to the general practitioner than to the oculist, on account of the connection between cerebro-spinal disease and paralysis of ocular muscles. The *post-mortem* in three cases showed that the nerve trunk itself was involved, which would point to an organic origin of those cases of periodic motor oculi paralysis, and I suppose in those cases relapses occurred from inflammatory exacerbations. I agree with Dr. Moyer that a large majority of the cases related would in all probability be functional. In his own case there were symptoms of migrain, viz., headache and vomiting accompanying the motor oculi paralysis. Organic lesions of the brain, such as basilar meningitis, etc., are in the great majority of cases, say 75 per cent., accompanied by optic neuritis. Since in the sixteen cases related by Dr. Moyer there is no mention, I believe, of optic neuritis I infer the motor oculi paralysis was not due to organic diseases of the brain, but to functional causes.

Dr. C. F. Sinclair: It has never been my experience to have met with a case precisely similar to that to which Dr. Moyer has referred, neither have I met with one in my reading. But it seems to me from the number of cases to which he refers, all with symptoms resembling one another in one or more particulars, and in all of which was found this element of periodicity, that we have almost data for the establishment of a new and definite pathological condition which would lead the way to very broad investigation in ophthalmic science. The paper is particularly interesting to me because one case to which reference has been made resembles in a very great degree one which came under my observation some weeks ago. It was a case of paresis of only one of the branches of the oculi motor nerve, but it was sufficiently marked to cause a high degree of ptosis. The element of periodicity was present in this case, and that, I take it, is the peculiar feature in the cases cited by Dr. Moyer. In the case to which I refer were associated with the paresis other peculiar and morbid conditions. My patient was a young woman, 17 years of age, of German parentage. When I first saw her I noticed at once a peculiar expression of the eyes, which I found came from the fact that one was a light, almost sky blue, and the other gray. She told me that three years previous both of her eyes had been dark brown and that two years ago the left eye began to grow lighter in color, changing to a light gray. That is a phenomenon which I must confess never to have heard of before, where the pigment in the eye of a person of that age has undergone such marked changes within a period of three years without any evidence of iritic inflammation to account for them. Dr. Moyer, I believe, mentions a case where this oculi motor paralysis had occurred in the spring successively on several different occasions. The attacks which my patient suffered occurred also in the spring, coming on each time in the same month and nearly the same day. When she came under my care she was suffering the third attack, which was precisely similar in every respect to the two preceding. There were several days of extreme languor and drowsiness, during which time there was an irresistible impulse to cry on the slightest occasion. Then followed the ptosis, accompanied with severe neuralgiac pain through the temple and over the vertex on one side, and at the same a small infiltrated ulcer made its appearance on the lower margin of the cornea. The usual local means I found entirely unavailing in checking the attack. This case suggested to my mind the possibility, and the suggestion has been renewed with added force to-night, that as ophthalmic surgeons we are too apt to dwell solely upon the local manifestations of disease in the arteries, tunics of the eye and its appendages and to limit our treatment entirely to local treatment. It seems to me that it is time for us to recognize the fact that in many of the morbid conditions of the eye which we have been accustomed to consider as altogether of local origin, the causation lies in reality much deeper, either in the great nerve centres or in diseased conditions of other and perhaps remote organs, and unless we direct our remedial measures to these our results can only be transient and unsatisfactory.

Dr. Moyer, in closing the discussion, said: Here, as in similar cases, we need to have an exact definition of what we mean by the term "functional." If we call only those lesions organic in which there is no absolute degeneration or destruction of the nerve cells, and speak of transitory congestion as "functional," then we must all admit that many, perhaps most, of these cases fall within the latter class. But the term used in its strictest sense—that is, in the sense that hysterical or reflex conditions are functional—then the only case, so far as our observation extends, ever reported is the one described by Jacobi. Every case of the seventeen so far reported, including our own, has been progressive. The recovery earlier in the disease was complete after each attack. Later a certain amount of paralysis would remain. Mobius refers the paralysis to an inflammation or congestion of the nucleus of the nerve, coming on periodically, and which in time results in a certain amount or even total paralysis. The anatomical diagnosis is the most interesting feature of the case. We have purposely refrained from offering any theories of our own regarding the probable seat or nature of the difficulty; yet we confess to a strong leaning toward the theory of muclear degenerative process. As Dr. Coleman has pointedly said, this disease is not associated with any changes in the retina or optic disc, which rather militates against the theory of a basilar meningetis as the basic pathological condition. We must, however, admit that the two earliest cases that came to a *post-mortem* examination were basilar in their nature, and in the third an enchondroma had impaired the integrity of the nerve.

